# Can Comprehensive Chromosome Screening Technology Improve IVF/ICSI Outcomes? A Meta-Analysis

**DOI:** 10.1371/journal.pone.0140779

**Published:** 2015-10-15

**Authors:** Minghao Chen, Shiyou Wei, Junyan Hu, Song Quan

**Affiliations:** 1 Department of Obstetrics and Gynecology, Reproductive Centre, Nanfang Hospital, Southern Medical University, Guangzhou, China; 2 Thoracic Department, West China Hospital, Sichuan University, Chengdu, China; 3 Emergency Department, The Third Affiliated Hospital of Guangzhou Medical University, Guangzhou, China; Institute of Zoology, Chinese Academy of Sciences, CHINA

## Abstract

**Objective:**

To examine whether comprehensive chromosome screening (CCS) for preimplantation genetic screening (PGS) has an effect on improving in vitro fertilization/intracytoplasmic sperm injection (IVF/ICSI) outcomes compared to traditional morphological methods.

**Methods:**

A literature search was conducted in PubMed, EMBASE, CNKI and *ClinicalTrials*.*gov* up to May 2015. Two reviewers independently evaluated titles and abstracts, extracted data and assessed quality. We included studies that compared the IVF/ICSI outcomes of CCS-based embryo selection with those of the traditional morphological method. Relative risk (RR) values with corresponding 95% confidence intervals (CIs) were calculated in RevMan 5.3, and subgroup analysis and Begg’s test were used to assess heterogeneity and potential publication bias, respectively.

**Results:**

Four RCTs and seven cohort studies were included. A meta-analysis of the outcomes showed that compared to morphological criteria, euploid embryos identified by CCS were more likely to be successfully implanted (RCT RR 1.32, 95% CI 1.18–1.47; cohort study RR 1.74, 95% CI 1.35–2.24). CCS-based PGS was also related to an increased clinical pregnancy rate (RCT RR 1.26, 95% CI 0.83–1.93; cohort study RR 1.48, 95% CI 1.20–1.83), an increased ongoing pregnancy rate (RCT RR 1.31, 95% CI 0.64–2.66; cohort study RR 1.61, 95% CI 1.30–2.00), and an increased live birth rate (RCT RR 1.26, 95% CI 1.05–1.50; cohort study RR 1.35, 95% CI 0.85–2.13) as well as a decreased miscarriage rate (RCT RR 0.53, 95% CI 0.24–1.15; cohort study RR 0.31, 95% CI 0.21–0.46) and a decreased multiple pregnancy rate (RCT RR 0.02, 95% CI 0.00–0.26; cohort study RR 0.19, 95% CI 0.07–0.51). The results of the subgroup analysis also showed a significantly increased implantation rate in the CCS group.

**Conclusions:**

The effectiveness of CCS-based PGS is comparable to that of traditional morphological methods, with better outcomes for women receiving IVF/ICSI technology. The transfer of both trophectoderm-biopsied and blastomere-biopsied CCS-euploid embryos can improve the implantation rate.

## Introduction

It has been thirty-seven years since the first IVF (in vitro fertilization) baby was born in 1978 [1]. In spite of recent advances, the majority of IVF cycles fail to achieve a live birth. One of the main causes of the depressing clinical outcomes has been proven to be embryo aneuploidy [2–4]. Aneuploidy is a very common abnormality in human embryos generated by IVF, particularly for women with advanced maternal age (AMA) [5, 6]. By the age of 40, it is not unusual for the proportion of aneuploid embryos to exceed 50% [7]. A high percentage of aneuploidy has also been found in the embryos of women with repeated implantation failure (RIF) [8, 9], repeated pregnancy loss (RPL) [10] and a partner with low sperm quality [11, 12]. Even for younger women (<35 years) with good prognosis, the aneuploidy rate remains high [13–15]. An aneuploid embryo is scarcely able to form a viable pregnancy. The high frequency of aneuploidy and its likely deleterious effects on embryo viability has led to the suggestion that embryos should be tested for chromosomal abnormalities before determining which ones to transfer to patients. Because traditional embryo selection methods based on morphology are incapable of detecting chromosomal abnormalities [16, 17], preimplantation genetic screening (PGS) was developed.

Fluorescence in situ hybridization (FISH) testing of a panel of chromosomes was previously the most widely applied method for aneuploidy screening. However, because previous data from random controlled trials with FISH-based PGS (PGS#1) showed no beneficial effects on live birth rates after IVF and even lower live birth rates for women with AMA [18, 19], the utilization of PGS#1 in attempts to improve IVF outcome has declined worldwide [20, 21]. The inefficiency of PGS#1 occurs for many reasons. One of the main limitations of FISH is that it can only test a restricted number of chromosomes in PGS. For embryos that are aneuploidy for untested chromosomes, FISH-based PGS cannot make an accurate evaluation. However, the objective of comprehensive chromosome screening (CCS) is to assess the entire chromosome complement (24 chromosomes).

Several studies have been conducted to assess the effect of CCS-based PGS on IVF/intracytoplasmic sperm injection (ICSI) outcomes, and 2 systematic reviews were published recently [22, 23]. However, these 2 systematic reviews did not conduct pooled analyses of the included studies. Therefore, we conducted a meta-analysis with more eligible studies to provide a more precise and comprehensive estimation of CCS-based PGS.

## Materials and Methods

### Literature search

We conducted electronic searches in the databases PubMed, EMBASE, CNKI (China National Knowledge Infrastructure) and *ClinicalTrials*.*gov* up to May 20, 2015 with no study design limitations and no language restrictions. The following search terms were used: ‘preimplantation genetic diagnosis’ or ‘PGD’ or ‘preimplantation genetic screening’ or ‘preimplantation test’ or ‘screening for aneuploidies’ or ‘embryo selection’ or ‘embryo screening’ and ‘comprehensive chromosomal screening’ or ‘CCS’ or ‘array comparative genomic hybridization’ or ‘array CGH’ or ‘aCGH’ or ‘single nucleotide polymorphism’ or ‘SNP’ or ‘quantitative real-time PCR’ or ‘qPCR’ or ‘next-generation sequencing’ or ‘NGS’. Moreover, a manual search of published articles was conducted to identify additional relevant studies.

### Study selection and data extraction

After duplicate publications were removed, two authors (CMH and WSY) independently examined the potentially relevant trials by checking the titles, abstracts and full-texts, and any problems of disagreement were resolved through group discussion. We adapted the preferred reporting items for systematic reviews and meta-analyses (PRISMA) flow-chart to depict the study selection process (see S1 File. PRISMA checklist. ) [24]. Published clinical trials were eligible for inclusion if they compared CCS-based PGS using blastocyst biopsy/trophectoderm biopsy to traditional morphological methods in genetically normal couples undergoing IVF and/or ICSI. Because polar body (PB) aneuploidy screening can only detect chromosomal abnormalities of meiotic origin and is limited to predicting subsequent embryo ploidy, we excluded studies associated with polar body biopsy to prevent selection bias. Then, two authors (CMH and WSY) independently extracted related information pertaining to the first author’s name, study design, year of publication, study period, geographic region, sample sizes of the groups using CCS versus morphological methods, type of CCS, patient characteristics, indication for PGS, day of biopsy, day of transfer, and fresh or frozen cycles. The primary outcome was the implantation rate per ET, and the secondary outcomes were the clinical pregnancy rate per cycle, ongoing pregnancy rate per cycle, live birth rate per cycle, miscarriage rate and multipregnancy rate. In all of the studies, the participants who underwent embryo biopsy for CCS-based PGS were defined as the CCS group, and the participants who used traditional morphological methods were defined as the control group.

### Quality assessment

Two reviewers (CMH and WSY) independently used the Newcastle-Ottawa Scale (NOS) to assess the quality of the included observational studies [25]. The NOS includes selection, comparability and outcome for cohort studies: 4 scores are assigned for the selection part, 2 scores for comparability and 3 scores for the outcome part. Studies with scores of 0 to 3, 4 to 6 and 7 to 9 were considered as low, moderate and high quality, respectively. The Cochrane Collaboration’s Handbook was used to assess the quality of the RCTs according to the following criteria: random sequence generation, allocation concealment, blinding, incomplete outcome data, selective outcome reporting and other potential sources of bias [26]. A judgment of ‘Yes’ indicates a low risk of bias, ‘No’ indicates a high risk of bias, and ‘Unclear’ indicates an unclear or unknown risk of bias.

### Statistical analysis

The effect of CCS-based PGS was assessed for the RCTs and cohort studies separately. We calculated the relative risk (RR) with corresponding 95% confidence intervals (CIs) for all of the outcomes reported in each study. A meta-analysis of the outcomes was performed when there were available data that could be combined and meta-analytical pooling was feasible. We assessed heterogeneity among the studies by conducting a standard Cochrane’s Q test with a significance level of α = 0.10. The I^2^ statistical test was performed to further examine heterogeneity. I^2^≥50% was considered to indicate substantial heterogeneity [27]. When heterogeneity existed, we attempted to identify potential sources of heterogeneity by examining individual studies and conducting subgroup analyses. Fixed-effect models were used to pool outcomes when heterogeneity among studies was considered to be statistically insignificant. Otherwise, a random-effect model was used to combine the results. Moreover, subgroup analyses were conducted according to study design, location, age of the participants, indication for PGS, stage of biopsy, platform for CCS and number of embryos transferred. Publication bias was estimated using Begg’s test [28]. A value of “Pr>|z|” greater than 0.05 for Begg’s funnel plots was considered to indicate negative publication bias. A one-way sensitivity analysis was performed to explore certain factors that would influence the effects. All of the statistics were two-tailed, and P<0.05 was considered statistically significant. RevMan 5.3 was used to perform the meta-analysis.

## Results

### Study selection and characteristics

**Fig 1:**
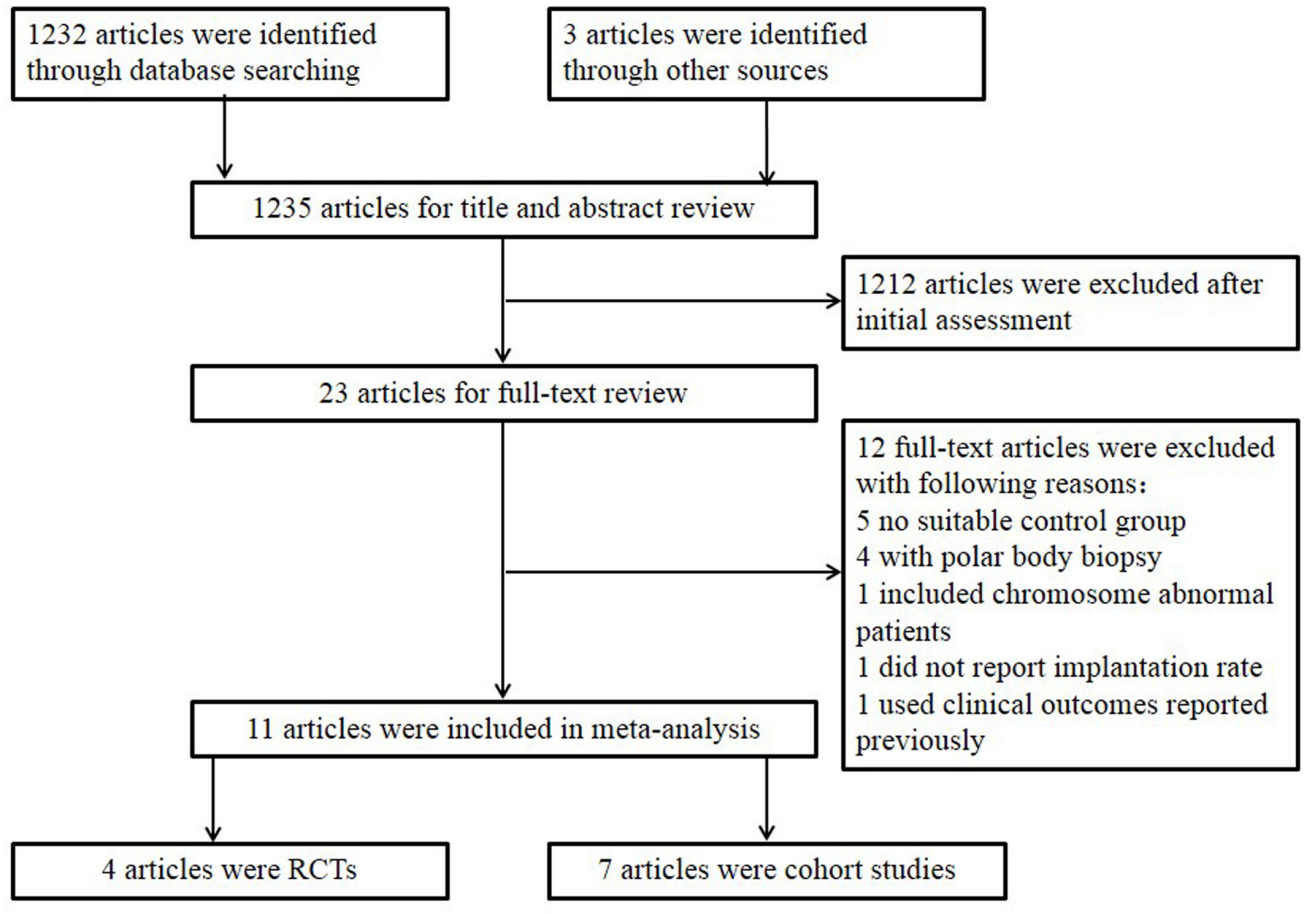
Flow chart of search and selection strategy.

A total of 1235 unduplicated titles and abstracts were identified in the initial search, and 23 articles were selected to undergo full-text assessment. Twelve studies did not fulfill the inclusion criteria, of these, five studies had no suitable control group, one study included chromosome abnornal patients, one study used clinical outcome data reported previously in another study we included in our meta-analysis, one study did not report implantation rate, four studies associated with polar body biopsy. Finally, 4 RCTs and 7 cohort studies that assessed the outcomes of CCS-based PGS versus traditional morphological-based selection in women undergoing IVF/ICSI met our inclusion criteria and were included in the meta-analysis [29–39]. A flow chart of the trials included in the meta-analysis is shown in Fig 1.

Overall, the 11 included studies accounted for 2425 ART cycles (RCTs: 247 for the CCS group, 255 for the control group; cohort studies: 729 for the CCS group, 1194 for the control group) in 2338 women, with ages ranging from 23 to 43 years. Of these studies, 8 were performed in North America, 2 in Europe [36, 37] and 1 in Asia [29]. For 3 RCTs, CCS-based PGS was performed for the women with good prognosis, and for 1 RCT [39] and all the cohort studies, CCS-based PGS was indicated in AMA, RIF, RPL, or for other reasons. The platform for CCS was CGH for 5 studies, qPCR for 3 studies, SNP for 1 study, and NGS for 1 study. Moreover, 3 studies performed embryo biopsy in the cleavage stage [29, 31, 37], and the others in the blastocyst stage. Single embryo transfer was performed in 3 studies [30, 32, 34], and 8 studies performed more than one embryo transfer. The main characteristics and quality features of the 4 RCTs and 7 cohort studies are shown in Tables 1 and 2.

**Table 1:** Characteristics of included RCTs.

Study and years	Patients (PGS/control)	Cycles (PGS/control)	Study design	Platform for PGS	Inclusion criteria	Exclusion criteria	ART and embryo transfer	Biopsy	Outcomes	Quality features
Yang 2012	Total 112 (56/56)	Total 103 (56/56)	Prospective randomized single-blind controlled trial	aCGH	<35 years; a history of regular ovulation; etiology of infertility was tubal factor or male factor (or both); no prior IVF treatment; no prior miscarriage; normal intrauterine contour; both ovaries intact; basal serum FSH<10 IU/l, basal estradiol<60 pg/ml.	Treatment incorporated donor gametes or frozen/thawed embryos.	ICSI; SET for both groups; all cycles involved day 6 blastocyst transfer; all cycles were fresh cycles.	Laser-assisted hatching for all 6–8 cleavage stage embryos on day 3; 3–5 herniated TE cells removed; biopsy on blastocysts on day 5.	Clinical pregnancy rate (same as implantation rate for SET); on-going pregnancy rate (more than 20 weeks GA); missed abortion rate.	Randomization by random number table; concealment of allocation not reported; single-blind; 2 centers; full paper; power calculation not reported; no intention to treat.
Schoolcraft 2012	Total 60 (30/30)	Total 60 (30/30)	IRB approved randomized control trial	SNP micro-array	>35 years	Exclusion criteria not reported.	ART method not reported; number of embryos transferred not reported; all cycles involved blastocyst transfer; frozen cycles for PGS group, fresh cycles for control group.	Biopsy method not reported; removed TE cells for biopsy.	Implantation rate; first trimester pregnancy lose rate.	Randomization by computer; concealment of allocation not reported; blinding not reported; one centre; only abstract; power calculation not reported; intention to treat.
Forman 2013	Total 175 (89/86)	Total 175 (89/86)	Randomized open label noninferiority trial	Real-time, polymerase chain reaction-based CCS.	<42 years; at most one prior failed IVF cycle; normal endometrial cavity; normal ovarian reverse (AMH>1.2 ng/mL, day 3 FSH<12 IU/L); at least 2 expanded blastocysts suitable for transfer or cryopreservation by day 6 of embryo development.	Severe male infertility; anovulatory polycystic ovarian syndrome; BMI>30 kg/m^2^.	ICSI; SET for CCS group, 2 blastocysts for control group. Fresh cycles involved day6 blastocyst transfer; frozen frozen cycles involved blastocyst transfer 5 days after starting P. Both groups involved fresh and frozen cycles.	Laser-assisted hatching for all cleaved embryos on day 3; number of TE cells removed not reported; biopsy on blastocysts on day 5.	Ongoing pregnancy rate; clinical miscarriage rate; sustained implantation rate; multiple pregnancy rate	Randomization by computer; concealment of allocation achieved by sequentially numbered, opaque, sealed envelopes; not blinded; 1 center; full paper; power calculation; intention to treat; block randomization used for fresh and frozen cycles.
Scott 2013	Total 155 (72/83)	Total 155 (72/83)	Prospective randomized controlled trial	Rapid quantitative real-time polymerase chain reaction (qPCR)-based comprehensive chromosome screening	21–42 years; no more than one prior failed IVF retrieval; normal endometrial cavity; basal FSH level<15IU/L; basal follicle cunt >8; available ejaculate sperm; willingness to limit transfer order to a maximum 2 embryos; 2 or more blastocysts by the afternoon of day 5.	Less than 2 blastocysts by day 5.	ICSI; maximum 2 embryos for each patients. CCS group involved blastocysts transfer on day 6; control group involved blastocysts on day 5; all cycles were fresh cycles.	Laser-assisted hatching for all embryos on day 3; number of TE cells removed not reported; biopsy on blastocysts on day 5.	Implantation rate; pregnancy rate; delivery rate.	Randomization by computer; concealment of allocation not reported; blind not reported; 1 center; full paper; power calculation; intention to treat; block randomization used for different age groups.

**Table 2:** Characteristics of included cohort studies.

Study and years	Patients (PGS/control)	Cycles (PGS/control)	Study design	Type of CCS used	Characteristics of CCS group patients	ART and embryo transfer	Biopsy	Outcomes	Confounders adjusted for	NOS score
Schoolcraft 2010	Total 119; (45/113)	Total 119; (45/113)	Prospective matched cohort study	Comparative genomic hybridization.	maternal age >35 years and/or with a history of unsuccessful IVF treatment or previous spontaneous abortion.	ICSI; maximum 4 for transfer in the CCS group; all cycles involved blastocyst transfer; all cycles were frozen cycles.	Laser; 3–10 TE cells(mean 5) removed; biopsy on expanding or expanded blastocysts on day 5 or day 6.	bHCG positive rate pre cycle; implantation rate (fetal sac); ongoing implantation rate (a fetus with heartbeat); Live birth rate	Year of treatment; one center; maternal age; day3 FSH level; day of transfer; number of previous unsuccessful IVF attempts.	9
Schoolcraft 2013	Total 737 (347/390)	Total 737 (347/390)	Prospective unmatched cohort study	CCS (method for CCS not reported).	The majority of female infertility presented with normal ovarian reserve (based on day 3 FSH, E2, antimullerian hormone, and antral follicle count). The majority of male infertility patients showed no indications of male-factor infertility (based on sperm concentration, motility, and strict Kruger morphology).	ART method not reported; SET for all groups; all cycles involved blastocyst transfer; only frozen cycles for PGS group; both frozen and fresh cycles for control group.	Biopsy method not reported; biopsy on blastocysts.	Implantation rate; missed abortion rate; ongoing pregnancy rate	One center.	7
Greco 2014	Total 121 (88/33)	Total 121 (88/33)	Prospective matched cohort study	Array CGH	<36 years; without a history of recurrent miscarriages; without abnormal karyotype, uterine abnormalities, autoimmune conditions, thrombophilia, severe endometriosis and reduced ovarian reserve; male patients without severe infertility (<500.000 motile sperm/mL after preparation) or high sperm DNA fragmentation; 43 couples with a history of 3–9 implantation failures, 45 couples underwent the first IVF attempt (good prognosis).	ICSI; SET for PGS group; 1–2 embryos for transfer in the control group; all cycles involved blastocyst transfer; included both frozen and fresh cycles in both groups.	Laser; 5–10 TE cells removed; biopsy on blastocysts on day 5 or day 6.	bHCG positive rate; implantation rate; clinical pregnancy rate; biochemical pregnancy rate; anembryonic pregnancy rate; tubal pregnancy rate; spontaneous abortion rate	Maternal age; day 3 FSH; day 3 AMH; antral follicle count; sperm count; sperm motility; sperm morphology; day of transfer; PGS patients divided into 2 subgroups (RIF PGS group and NO RIF PGS group)	8
Keltz 2013	Total 346 (35/311)	Total 433 (39/394)	Retrospective unmatched cohort study	Array CGH	At least of 5 embryos six or more cells on day 3; indication for PGS included advanced maternal age, RIF, RPL.	ICSI; generally maximum 1 embryo for transfer for patients <35 years, maximum 2 for patients >35years; PGS group involved only blastocyst transfer; all cycles were fresh cycles.	Laser; single blastomere removed; biopsy on cleavage-stage embryos on day 3.	Implantation rate; clinical pregnancy rate; ongoing pregnancy rate; multiple-pregnancy rate; miscarriage rate (prior to 20 gestational weeks)	Year of treatment; one center; number of healthy-appearing embryos on day 3; sub-analysis for maternal age (<35years or >35years).	9
Wang 2014	Total 54 (25/29)	Total 54 (25/29)	Prospective unmatched cohort study	Array CGH	2 or more spontaneous abortions; without abnormal karyotype, uterine abnormalities, autoimmune conditions, severe endometriosis and reduced ovarian reserve; no indications of male-factor infertility.	ICSI; maximum 2 embryos for transfer in PGS group, maximum 3 in the control group; all cycles involved blastocyst transfer; all cycles were frozen cycles.	Laser; 1–2 blastomeres removed; biopsy on cleavage-stage embryos on day 3.	Implantation rate; clinical pregnancy rate; first trimester abortion rate.	Year of treatment; one center.	7
Forman 2012	Total 322 (140/182)	Total 322 (140/182)	Retrospective matched cohort study	Quantitative real-time PCR (qPCR).	Had four or more mature follicles (>14mm) on the day of hCG administration. Indication for PGS: advanced maternal age (>35 years); had a previous failed IVF cycle; had a history of recurrent pregnancy loss; wanted to optimize outcomes with SET.	ICSI for PGS group; SET for both groups; all cycles involved blastocyst transfer; included both frozen and fresh cycles in both groups.	Laser; about 5 TE cells removed; biopsy on blastocysts on day 5.	Chemical preganncy rate; ongoing pregnancy rate; clinical pregnancy rate; monozygotic twin rate; gestational age at delivery; birthweight.	Maximal Day 3 FSH; prior deliveries; prior COH/IUI cycles; prior FETs.	7
Lukaszuk 2014	Total 98 (45/53)	Total 98 (45/53)	Prospective matched cohort study	Semiconductor—based next-generation sequencing (NGS)	Repeated implantation failures (more than 2 previous unsuccessful failures).	ICSI; minimum 1 embryo for transfer in both groups; all cycles involved blastocyst transfer; all cycles were fresh cycles.	Laser; single blastomere removed; biopsy on cleavage-stage embryos on day 3.	Clinical pregnancy rate (per cycle; per ET); implantation rate; multiple pregnancy rate; ectopic pregnancy rate; OHSS rate; biochemical pregnancy rate; spontaneous abortion rate; ongoing pregnancy rate; live birth rate.	Year of treatment; one center; infertility etiology; number of failed cycles; duration of infertility; maternal age; BMI; antral follicle count; range of hormonal and other prognostic markers (AMH, inhibin B, basal FSH, basal LH, basal E2, DHEAS, testosterone, SHBG).	9

### Quality assessment

Study design and methodological quality varied among the 4 RCTs. One study used a random number table to generate a randomized sequence [32]; 1 study used computer-generated randomization [35]; randomization was stratified by age group in 1 study [33] and the remaining study did not explicitly describe sequence generation [39]. Adequate measures of allocation concealment were used and explicitly described in only one study [35]. Single blinding was performed in one study [32], 1 study was not blinded [35], and the remaining 2 studies did not describe the method of blinding [33, 39].

For the 7 cohort studies that were included, the NOS scores ranged from 7 to 9, with a mean score of 8. All of the studies provided information on the populations in the CCS group and the control group. However, only 4 studies were well matched between the CCS group and the control group [38, 36, 31, 37], and the other 3 studies were unmatched [29, 30, 34]; thus, comparability bias might exist in the 3 studies because important factors that could influence the results were not controlled for. The follow-up period for outcome was adequate for all of the studies, and the outcome measurement was objective.

### Main outcomes

#### Implantation rate

**Fig 2:**
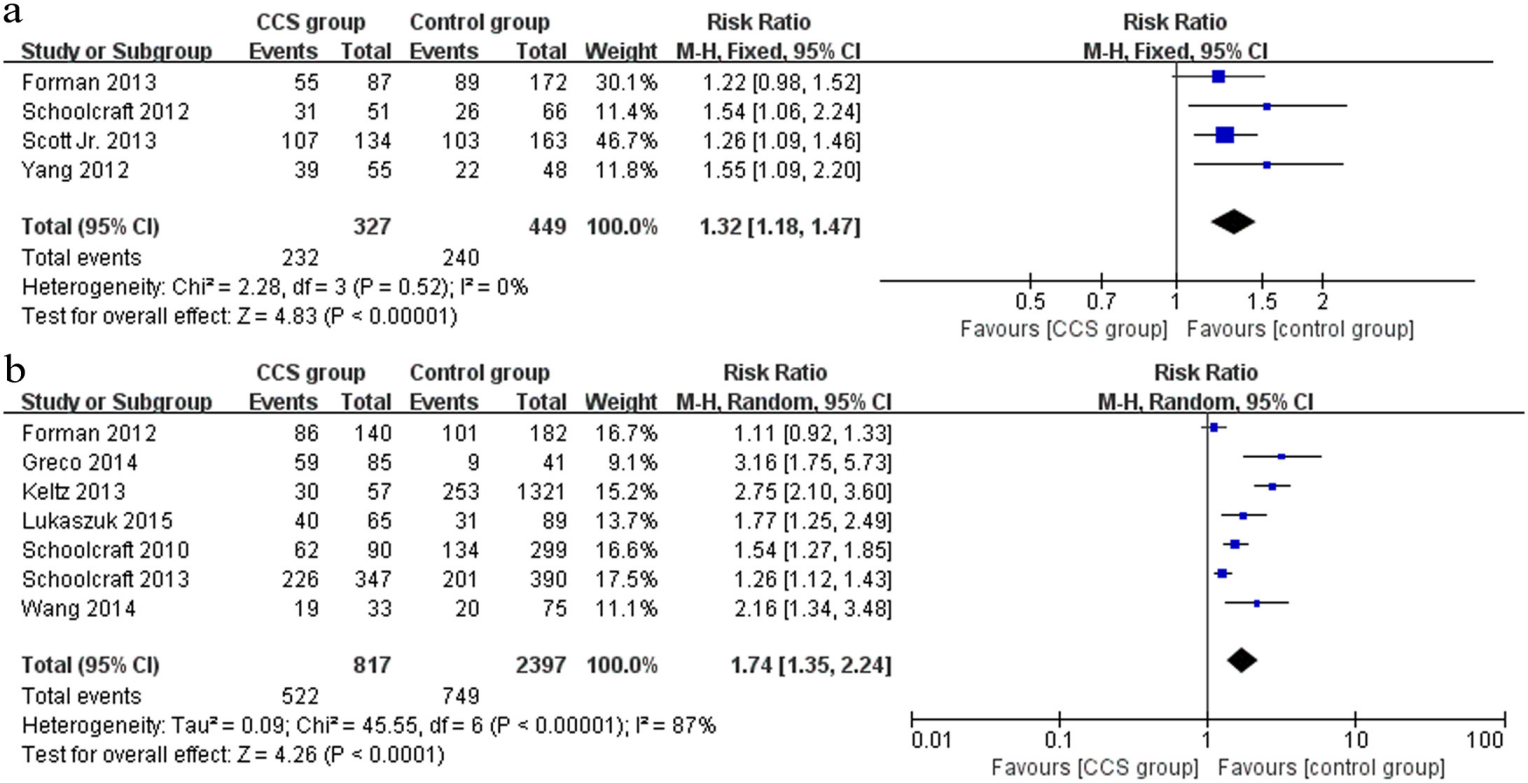
Forest plots showing the results of meta-analysis on implantation comparing the effect of CCS-based PGS and traditional morphological method after IVF/ICSI. (a) Forest plot of pooled RR on implantation of RCTs; (b) Forest plot of pooled RR on implantation of cohort studies.

All of the included studies (4 RCTs and 7 cohort studies) provided data on the implantation rate. Within the 4 RCTs (n = 776) that compared CCS-based PGS and traditional morphological-based selection, the CCS group showed a higher implantation rate than the control group (RR 1.32, 95% CI 1.18–1.47) (Fig 2a). The same effects were observed within the 7 cohort studies (n = 3214) (RR 1.74, 95% CI 1.35–2.24) (Fig 2b).

#### Clinical pregnancy

**Fig 3:**
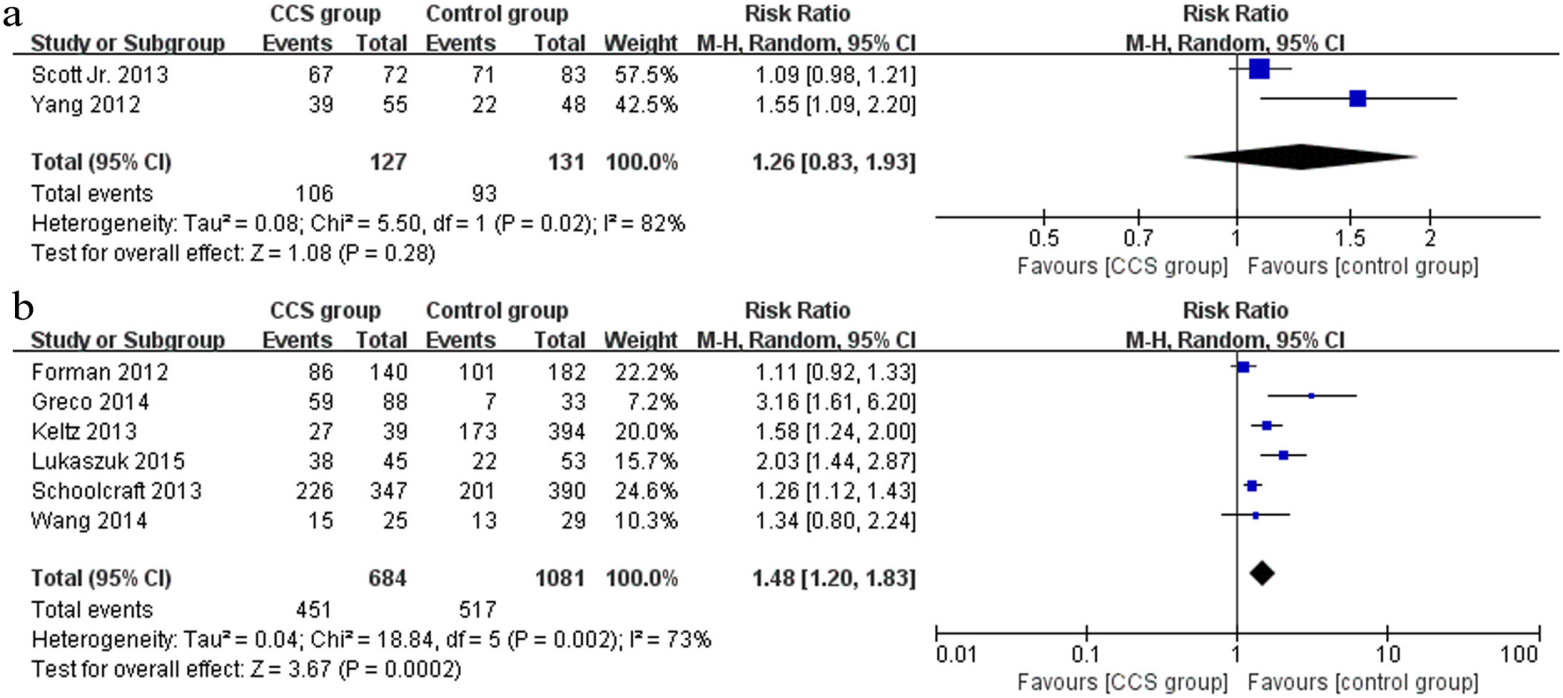
Forest plots showing the results of meta-analysis on clinical pregnancy comparing the effect of CCS-based PGS and traditional morphological method after IVF/ICSI. (a) Forest plot of pooled RR on clinical pregnancy of RCTs; (b) Forest plot of pooled RR on clinical pregnancy of cohort studies.

Eight studies (2 RCTs and 6 cohort studies) reported clinical pregnancy outcomes. The outcome from the pooled analysis of 2 RCTs [33, 32] (n = 258) showed a non-significant effect between the CCS group and the control group (RR 1.26, 95% CI 0.83–1.93) (Fig 3a), whereas a statistically significant increase in clinical pregnancy rate because of CCS-based PGS was observed in 6 cohort studies [29, 36, 30, 31, 37, 34] (n = 1765) (RR 1.48, 95% CI 1.20–1.83) (Fig 3b).

#### Ongoing pregnancy

**Fig 4:**
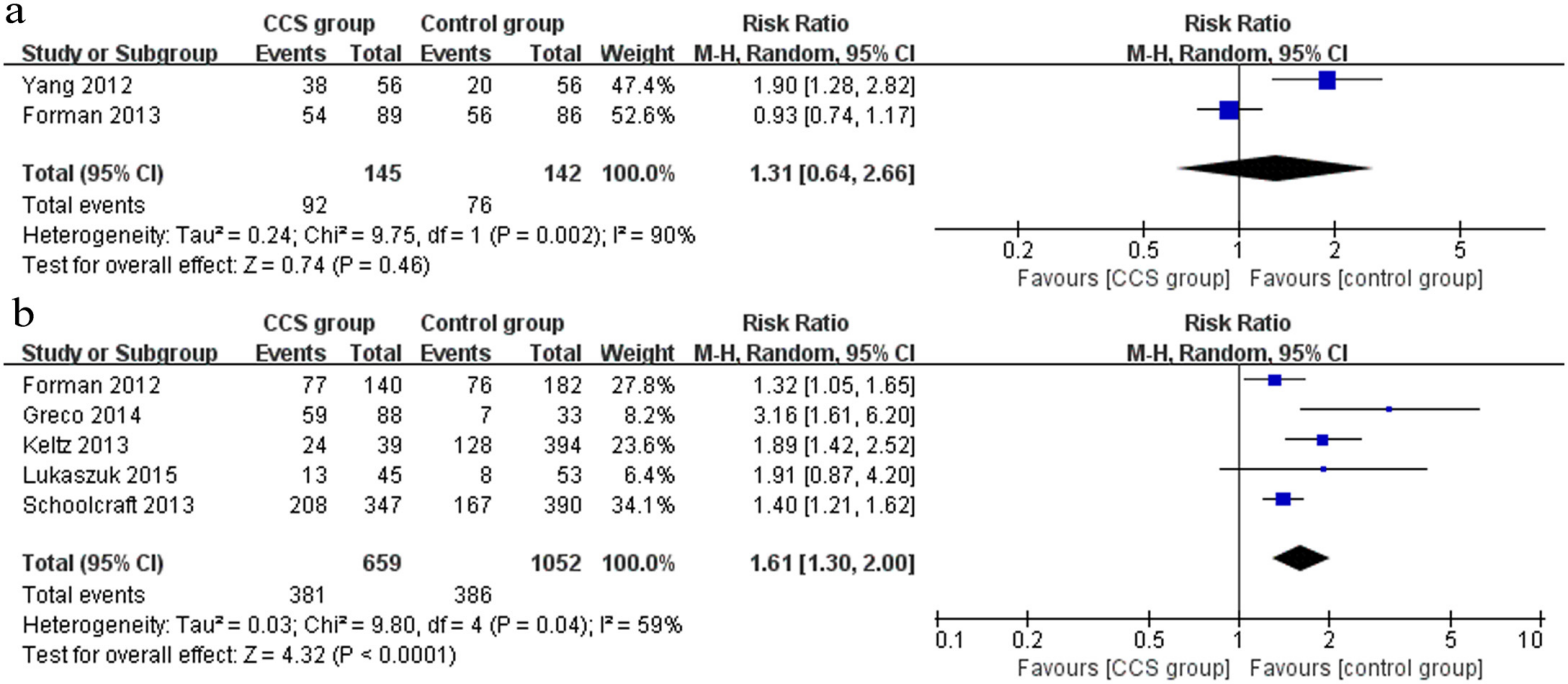
Forest plots showing the results of meta-analysis on ongoing pregnancy comparing the effect of CCS-based PGS and traditional morphological method after IVF/ICSI. (a) Forest plot of pooled RR on ongoing pregnancy of RCTs; (b) Forest plot of pooled RR on ongoing pregnancy of cohort studies.

Seven studies (2 RCTs and 5 cohort studies) presented data regarding ongoing pregnancy. The pooled ongoing pregnancy rate appeared to be higher in the CCS group than in the control group in 2 RCTs [35, 32] (n = 287), but there was no significant difference between the two groups (RR 1.31, 95% CI 0.64–2.66) (Fig 4a). However, the pooled outcome of 5 cohort studies [36, 30, 31, 37, 34] (n = 1711) showed that CCS significantly improved the ongoing pregnancy rate (RR 1.61, 95% CI 1.30–2.00) (Fig 4b).

#### Live birth

**Fig 5:**
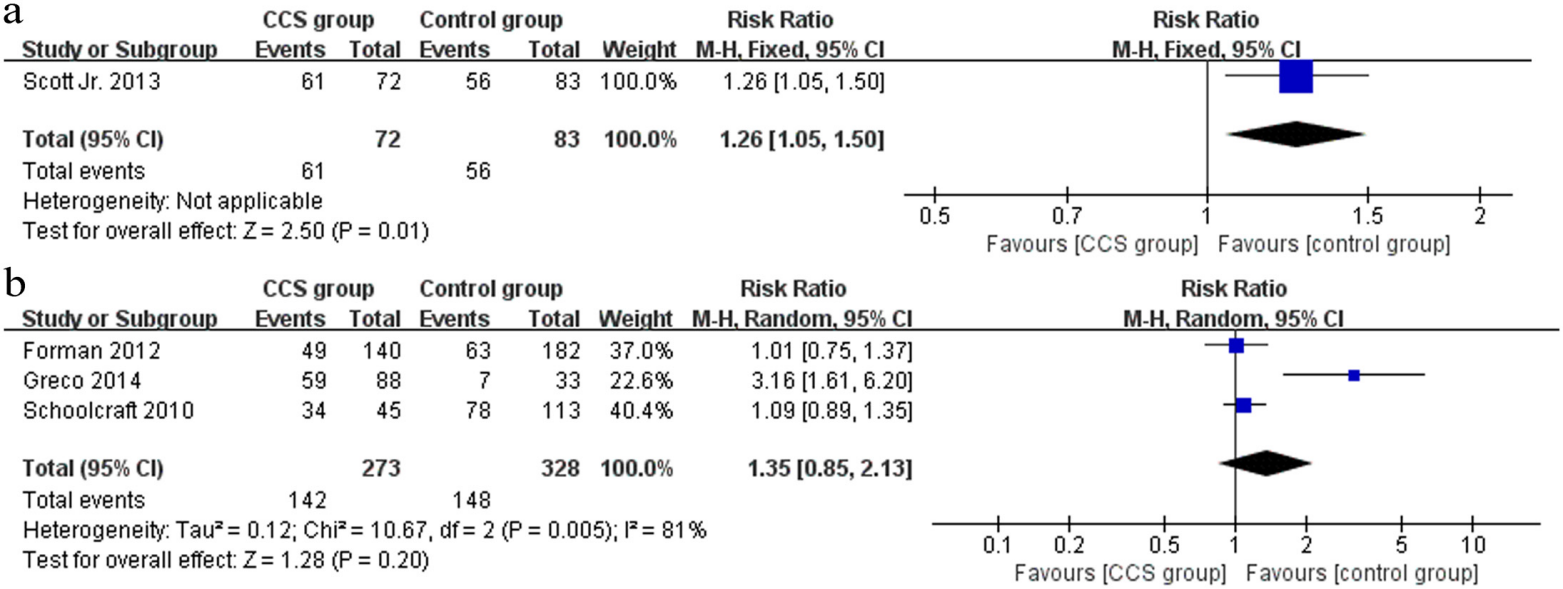
Forest plots showing the results of meta-analysis on live birth comparing the effect of CCS-based PGS and traditional morphological method after IVF/ICSI. (a) Forest plot of pooled RR on live birth of RCTs; (b) Forest plot of pooled RR on live birth of cohort studies.

A total of 4 studies (1 RCT and 3 cohort studies) investigated the outcome of live birth. In the RCT [33], there was a statistically significantly higher live birth rate in the CCS group (61/72) compared to the control group (56/83) (RR 1.26, 95% CI 1.05–1.50) (Fig 5a). However, when the outcome was pooled for the 3 cohort studies [38, 36, 34] (n = 601), no significant difference in live birth rate was observed between the CCS group and the control group (RR 1.35, 95% CI 0.85–2.13) (Fig 5b).

#### Miscarriage rate

**Fig 6:**
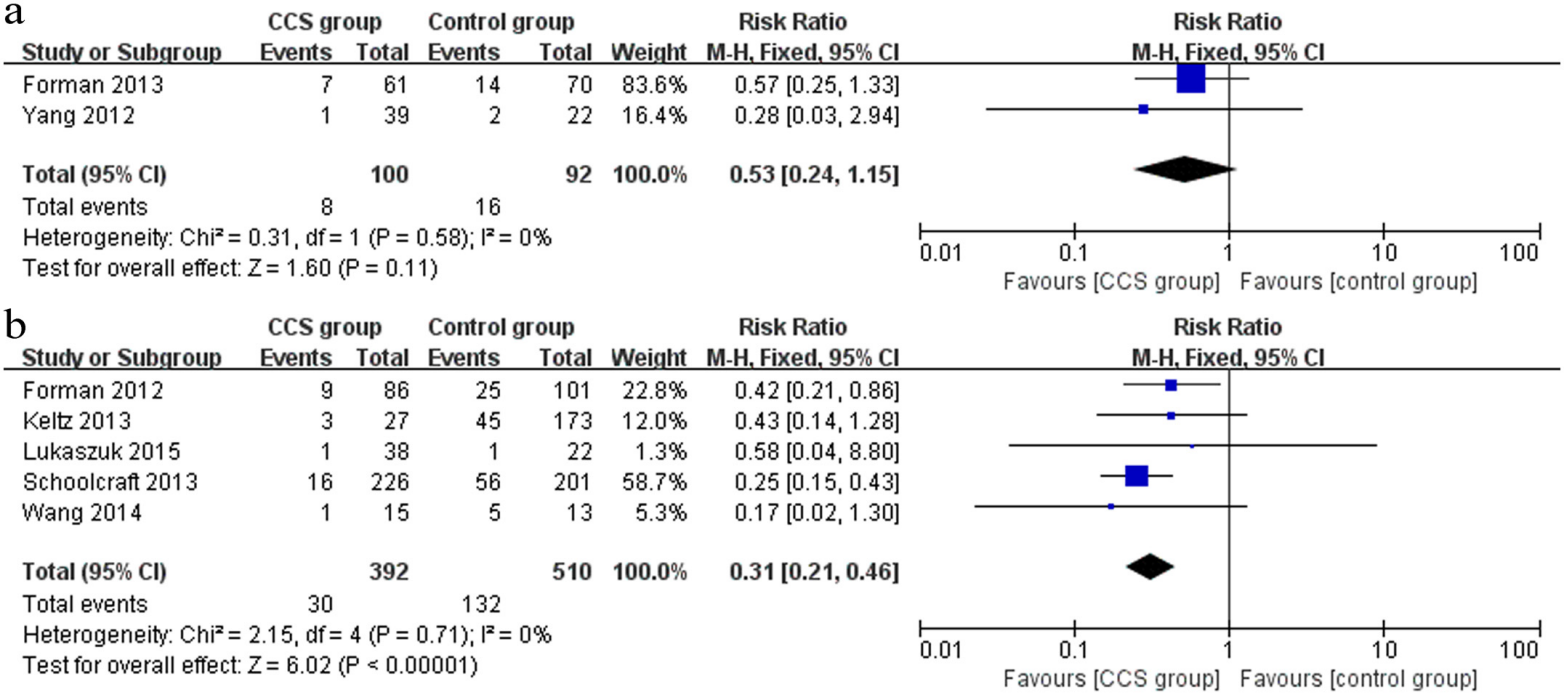
Forest plots showing the results of meta-analysis on miscarriage comparing the effect of CCS-based PGS and traditional morphological method after IVF/ICSI. (a) Forest plot of pooled RR on miscarriage of RCTs; (b) Forest plot of pooled RR on miscarriage of cohort studies.

Two RCTs [35, 32] and 5 cohort [29–31, 37, 34] studies evaluated the outcome of miscarriage between the CCS group and the control group. The pooled outcome from 2 RCTs (n = 192) showed a decreased miscarriage rate in the CCS group, but there was no significant difference between the two groups (RR 0.53, 95% CI 0.24–1.15) (Fig 6a). Nevertheless, a pooled analysis of outcome including the 5 cohort studies (n = 902) showed that the miscarriage rate was significantly lower in the CCS group (RR 0.31, 95% CI 0.21–0.46) (Fig 6b).

#### Multiple pregnancy

**Fig 7:**
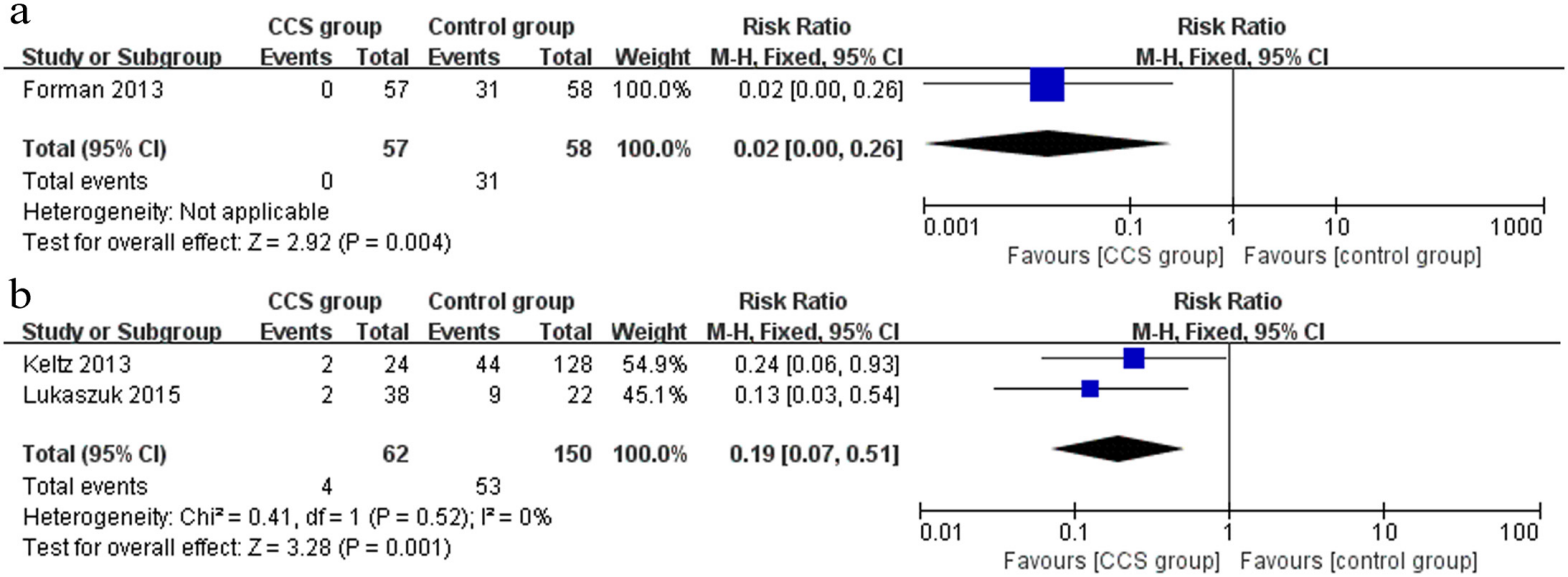
Forest plots showing the results of meta-analysis on multiple pregnancy comparing the effect of CCS-based PGS and traditional morphological method after IVF/ICSI. (a) Forest plot of pooled RR on multiple pregnancy of RCTs; (b) Forest plot of pooled RR on multiple pregnancy of cohort studies.

Only 3 studies (1 RCT and 2 cohort studies) reported multiple pregnancy rate data. In the RCT [35], the multiple pregnancy rate was significantly lower in the CCS group (0/57) than in the control group (31/58) (RR 0.02, 95% CI 0.00–0.26) (Fig 7a). Moreover, the same effect was observed in the pooled outcome of the 2 cohort studies [31, 37] (RR 0.19, 95% CI 0.07–0.51) (Fig 7b).

A summary of the results of the meta-analysis comparing CCS with no CCS outcomes in the included RCTs and cohort studies is presented in Table 3.

**Table 3:** Summary of results of meta-analysis of CCS compared with no CCS outcomes in included RCTs and cohort studies.

Outcome		No. of participants or cycles (trials)	CCS group	Control group	Pooled effct RR (95% CI)	Analysis model	Heterogeneity (I^2^)
Implantation rate	RCTs	776 (4)	232/327	240/449	1.32 (1.18, 1.47)	Fixed	0%
	Cohort studies	3214 (7)	522/817	749/2397	1.74 (1.35, 2.24)	Random	87%
Clinical pregnancy	RCTs	258 (2)	106/127	93/131	1.26 (0.83, 1.93)	Random	82%
	Cohort studies	1756 (6)	451/684	517/1081	1.48 (1.20, 1.83)	Random	73%
Ongoing pregnancy	RCTs	287 (2)	92/145	76/142	1.31 (0.64, 2.66)	Random	90%
	Cohort studies	1711 (5)	381/659	386/1052	1.61 (1.30, 2.00)	Random	59%
Live birth	RCTs	155 (1)	61/72	56/83	1.26 (1.05, 1.50)	Fixed	NA
	Cohort studies	601 (3)	142/273	148/328	1.35 (0.85, 2.13)	Random	81%
Miscarriage rate	RCTs	192 (2)	8/100	16/92	0.53 (0.24, 1.15)	Fixed	0%
	Cohort studies	902 (5)	30/392	132/510	0.31 (0.21, 0.46)	Fixed	0%
Multiple pregnancy	RCTs	115 (1)	0/57	31/58	0.02 (0.00, 0.26)	Fixed	NA
	Cohort studies	212 (2)	4/62	53/150	0.19 (0.07, 0.51)	Fixed	0%

#### Heterogeneity and subgroup analysis

The heterogeneity in the pooled risk estimates of our outcomes ranged from an I^2^ test result of 0 to 90% for both the RCTs and the cohort studies. Therefore, we performed a subgroup analysis for both the RCTs and the cohort studies addressing factors that might result in heterogeneity: age, location of the study, indication for CCS, day of biopsy, platform for CCS, number of embryos transferred and study design. We did not perform the subgroup analysis for outcomes other than the implantation rate because of the low number of related reports. As shown in Table 4, the implantation rate was higher in the CCS group than in the control group in any individual subgroup, and except for the pooled effect of the 2 retrospective cohort studies showing no significant differences between the CCS group and the control group, there were significant differences in the implantation rate in all of the other pooled subgroup analyses. The subgroup analysis results showed that the heterogeneity values were not substantially changed by the factors mentioned above.

**Table 4:** Subgroup analysis outcomes.

	Subgroup	Outcome	No. of studies	CCS group Implantated/Transferred embryos	Control group Implantated/Transferred embryos	Pooled effct RR (95% CI)	Analysis model	Heterogeneity (I^2^)
RCTs	Age	<35y	3	201/276	214/383	1.29 (1.15, 1.45)	Fixed	0%
		>35y	1	31/51	26/66	1.45 (1.06, 2.24)	Fixed	NA
	Indications for CCS	Good prognosis	3	201/276	214/383	1.29 (1.15, 1.45)	Fixed	0%
		AMA	1	31/51	26/66	1.45 (1.06, 2.24)	Fixed	NA
	Platform for PGS	aCGH	1	39/55	22/48	1.55 (1.09, 2.20)	Fixed	NA
		qPCR	2	162/221	192/335	1.25 (1.10, 1.41)	Fixed	0%
		SNP	1	31/51	36/66	1.54 (1.06, 2.24)	Fixed	NA
Cohort studies	Study design	Prospective studies	5	406/620	395/894	1.70 (1.33, 2.17)	Random	75%
		Retrospective studies	2	116/197	354/1503	1.73 (0.71, 4.24	Random	97%
	Age	<35y	2	99/150	40/130	2.25 (1.25, 4.06)	Random	67%
		>35y	4	404/634	689/2192	1.53 (1.13, 2.08)	Random	91%
	Location	North America	4	404/634	689/2192	1.52 (1.13, 2.08)	Random	91%
		Europe	2	99/150	40/130	2.25 (1.25, 4.06)	Random	67%
		Asia	1	19/33	20/75	2.16 (1.34, 3.48)	Random	NA
	Indications for CCS	AMA, RIF, RPL or other	4	404/634	689/2192	1.52 (1.13, 2.08)	Random	91%
		RIF	2	68/106	40/130	2.21 (1.27, 3.84)	Random	61%
		RPL	1	19/33	20/75	2.16 (1.34, 3.48)	Random	NA
	Stage of biopsy	Blastocyst stage biopsy	4	433/662	445/912	1.42 (1.12, 1.79)	Random	80%
		Cleavage stage biopsy	3	89/155	304/1485	2.23 (1.66, 2.99)	Random	52%
	Platform for PGS	aCGH	4	170/265	416/1736	2.23 (1.53, 3.27)	Random	81%
		NGS	1	40/65	31/89	1.77 (1.25, 2.49)	Random	NA
		qPCR	1	86/140	101/182	1.11 (0.92, 1.33)	Random	NA
	Embryo transfer	Single embryo transfer	2	312/487	302/572	1.20 (1.06, 1.36)	Random	27%
		More than one embryo transfer	5	210/330	447/1825	2.11 (1.57, 2.83)	Random	74%

#### Sensitivity analysis and publication bias

**Fig 8:**
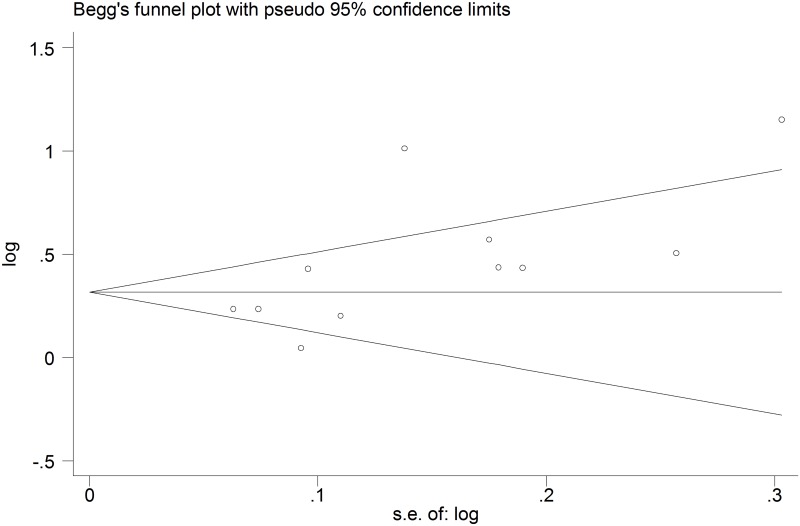
Begg’s funnel plot for assessment of publication bias, suggesting no significant small-study bias.

The pooled effect results remained practically unchanged when we performed a one-way sensitivity analysis. Begg’s test did not show significant small-study bias (p = 0.062) (Fig 8).

## Discussion

To review the current literature regarding the use of CCS technology for PGS and embryo selection and to assess the effect of CCS on the clinical outcomes of IVF, we included 11 studies (4 RCTs and 7 cohort studies) accounting for 2425 ART cycles in 2338 women to conduct a meta-analysis. Our results showed that CCS-based PGS was statistically significantly associated with an increased implantation rate (RCT-based RR 1.32, 95% CI 1.18–1.47; cohort study RR 1.74, 95% CI 1.35–2.24). The RR value demonstrated that there is great potential benefit from using CCS-based PGS over traditional morphological methods. And CCS-based PGS was also related to increases in the clinical pregnancy rate, ongoing pregnancy rate, and live birth rate, and decreases in the miscarriage and multipregnancy rates. To our knowledge, two systematic reviews evaluating the clinical effectiveness of CCS-based PGS have been published recently [23, 22]. Dahdouh’s study [23], which included only 3 RCTs on good prognosis patients, found that when the same number of embryos were transferred, CCS-based PGS was associated with a higher implantation rate and clinical pregnancy rate. The literature search in Lee’s systematic review [22] included the same 3 RCTs and 16 observational studies, and in addition to the trials assessing trophectoderm biopsy and blastomere biopsy, their study also included trials using PB biopsy for CCS. Our results were mainly in line with those of the systematic reviews, but this is the first meta-analysis to compare CCS-based embryo selection to traditional morphological methods, and we evaluated more outcomes including the live birth rate and multipregnancy rate.

In our review, all of the included trials were considered to be at low to moderate risk of bias. For the 4 RCTs included, one was presented as an abstract. Two studies provided information about random sequence generation. Most of the trials did not provide detailed information on allocation concealment or the method of blinding; however, blinding is not likely to influence outcome judgment or measurement. Three RCTs reported no loss to follow-up, and intention-to-treat analysis was performed for these studies. In one study, 9 patients were missing for various reasons, but intention-to-treat analysis was conducted. For the 7 included cohort studies (5 prospective studies and 2 retrospective studies), selection bias was not thought to play a major role, but comparability bias might exist among these studies.

The effect of CCS-based PGS on the implantation rate for patients with different indications for PGS and from different regions was also analyzed. The results of Dahdouh’s systematic review [23] revealed that the use of CCS-based PGS improved the implantation rate in good prognosis patients, and the results of our meta-analysis revealed that CCS-based PGS can also benefit patients with AMA, RIF and RPL. Because most of the patients involved in our study presented with normal ovarian reserves and no male factor infertility, whether CCS-based PGS can also benefit these patients, particularly for women who do not have an abundance of embryos to evaluate, remains to be determined. No statistically significant difference was found in the subgroup analysis conducted by patient location.

There are 3 possible sources of material for PGS testing: the first and second polar bodies, 1 or 2 blastomere biopsy from cleavage-stage embryos and 5–10 trophoblast cell biopsy from blastocysts. The accuracy of PB testing is significantly lower than cleavage stage blastomere testing or the trophoblast cell analysis, primarily because of the inability of this method to comprehensively assess all origins of embryonic aneuploidy [40, 41]. Regarding the advantages of cleavage stage biopsy versus blastocyst biopsy, recent studies found that a day 3 biopsy decreased the blastocyst rate and the implantation rate whereas a blastocyst biopsy showed no effect on embryonic developmental competence [35, 31, 42, 43]. Another advantage of blastocyst biopsy is the ability to test 5–10 trophectoderm cells, resulting in greater efficiency and a lower no-result rate [44]. Based on data procured from observational studies included in our meta-analysis, cleavage stage PGS was also feasible for CCS, and it showed an even better implantation outcome than blastocyst stage PGS (RR 2.23, 95% CI 1.66–2.99 vs. RR 1.42, 95% CI 1.12–1.79). However, further high quality evidence derived from randomized control trials is still required to determine the best time for CCS biopsy.

CCS-based PGS can be performed through a wide variety of methods. For DNA amplification, the available methods include multiple displacement amplification (MDA) [45–49], PCR (polymerase chain reaction) [50–52], and targeted multiplex PCR [53, 54]. For evaluating the amplified DNA, methods include CGH (comparative genetic hybridization) arrays [55–57, 36, 31, 38, 29, 32], SNP (single nucleotide polymorphism) arrays [48, 49, 51, 52, 58], NGS (next-generation sequencing)-based CCS [59, 60, 37] and qPCR-based CCS [61, 62, 54, 33, 34]. Among these methods, array CGH was the first technology to be widely used and has been validated by testing cells of a known genotype [63]. Comparing to other methods, NGS may offer more potential advantages including lower cost, reduced time and higher chromosomal analysis resolution [56, 64]. Moreover, the equivalence of NGS-based CCS to array CGH in the detection of chromosomal aneuploidy has been demonstrated [65, 66]. Most of the studies included in our analysis used aCGH and qPCR for karyotype screening. The only cohort study that used qPCR showed no significant effect on the implantation rate between the CCS group and the control group, and pooled data from the other separate PGS platform subgroup analyses all showed improvements in the implantation outcome.

One of the main targets of CCS-based PGS is to increase the number of singleton deliveries, which are considered the ideal outcome of IVF. Two RCTs and 2 cohort studies included in our meta-analysis transferred single embryos for both groups, and the other 2 RCTs and 5 cohort studies transferred significantly more embryos for the control group than the CCS group. According to our results, CCS improved the implantation rate even when fewer embryos were transferred.

Generally, the selection of embryos for transfer is based on traditional morphological assessment alone. Nonetheless, this method is not efficient enough because on average, only 1 in 4 treatments results in successful implantation. The development of alternative methods for diagnosing embryo viability preimplantation brings hope for improving the time to pregnancy and facilitating eSET. Other technologies for evaluating embryos include PGS, time-lapse microscopy, embryo proteomics and metabolomics. Time-lapse systems can take digital images at frequent time intervals without removing the embryos from the incubator. In recent years, algorithms based on morphokinetic parameters obtained through time-lapse methods have been created to predict the competence of embryo development, but until now, evidence of significant differences in clinical outcomes has been insufficient to choose between time-lapse systems and conventional incubation [67–69]. Some recent studies have tried to correlate time-lapse morphokinetic parameters with aneuploidy, but the results of these studies indicated that the selection of embryos by time-lapse cannot yet replace PGS [70–72]. Proteomics and metabolomics are typically able to characterize thousands of proteins and metabolites reflected the physiological status of embryos. Although studies have reported positive results regarding correlations between metabolic status and embryo developmental competence, the available clinical data on IVF outcomes still lack support for the use of proteomics or metabolomics [73–77].

The traditional morphological method prevents damage caused by the biopsy procedure and cuts the cost of genetic testing while having the potential to provide good cumulative pregnancy and live birth rates. However, the transfer of aneuploid embryos may result in miscarriage, and repeated transfer cycles not only cause emotional stress but also result in extra costs, including the costs of repeated endometrial preparation, monitoring scans and working days loss; such costs can exceed the euploidy testing cost. Although not all euploid embryos detected by CCS-based PGS can lead to successful implantation, but at present, CCS-based PGS appears to be the most reliable method for diagnosing preimplantation embryo viability. With the development of NGS, the cost of PGS may decline significantly, allowing greater access for more patients. Better designed randomized controlled trials are required to provide sufficient evidence regarding the efficiency of CCS-based PGS and to compare this technology to other methods used to evaluate preimplantation embryo viability.

## Supporting Information

S1 File PRISMA checklist. (DOC)
