# Supplementary material for: Can Comprehensive Chromosome Screening Technology Improve IVF/ICSI Outcomes? A Meta-Analysis
Source: PLoS One. 2015 Oct 15;10(10):e0140779. doi: 10.1371/journal.pone.0140779 (PMC4607161; doi:10.1371/journal.pone.0140779)
Supplement: S1 File — (DOC) [file pone.0140779.s001.doc]

| **Section/topic** | **#** | **Checklist item** | **Reported on page #** |
| --- | --- | --- | --- |
| **TITLE** | | |  |
| Title | 1 | Can comprehensive chromosome screening technology improve the IVF/ICSI outcomes? A meta-analysis. | 1 |
| **ABSTRACT** | | |  |
| Structured summary | 2 | ***Objetive*:** To examine whether comprehensive chromosome screening (CCS) for preimplantation genetic screening (PGS) has an effect on improving in vitro fertilization/intracytoplasmic sperm injection (IVF/ICSI) outcomes compared to traditional morphological methods.  ***Data sources*:** A literature search was conducted in PubMed, EMBASE, CNKI and *ClinicalTrials.gov* up to May 2015. A manual search was conducted in published articles in order to identify additional relevant studies.  ***Study selection*:** Two reviewers independently evaluated titles and abstracts, extracted data and assessed quality. We included studies that compared the IVF outcomes of CCS-based embryo selection to traditional morphological method. For all included studies, implantation rate per ET was defined as primary outcome, and secondary outcomes consisted of clinical pregnancy rate per cycle, ongoing pregnancy rate per cycle, live birh rate per cycle, miscarriage rate and multipregnancy rate.  ***Synthesis methods*:** Relative risk (RR) with corresponding 95% confidence intervals (CIs) were calculated in RevMan 5.3. And subgroup analysis and Begg’s test were conducted for the source of heterogeneity and potential publication bias, respectively.  ***Results*:** Four RCTs and seven cohort studies were included. A meta-analysis of the outcomes showed that compared to morphological criteria, euploid embryos identified by CCS were more likely to be successfully implanted (RCT RR 1.32, 95% CI 1.18-1.47; cohort study RR 1.74, 95% CI 1.35-2.24). CCS-based PGS was also related to an increased clinical pregnancy rate (RCT RR 1.26, 95% CI 0.83-1.93; cohort study RR 1.48, 95% CI 1.20-1.83), an increased ongoing pregnancy rate (RCT RR 1.31, 95% CI 0.64-2.66; cohort study RR 1.61, 95% CI 1.30-2.00), and an increased live birth rate (RCT RR 1.26, 95% CI 1.05-1.50; cohort study RR 1.35, 95% CI 0.85-2.13) as well as a decreased miscarriage rate (RCT RR 0.53, 95% CI 0.24-1.15; cohort study RR 0.31, 95% CI 0.21-0.46) and a decreased multiple pregnancy rate (RCT RR 0.02, 95% CI 0.00-0.26; cohort study RR 0.19, 95% CI 0.07-0.51). The results of subgroup analysis based on day for biopsy, indication for CCS, number of embryos transferred, age and geography all shown a significantly increased implantation rate in the CCS group.  ***Conclusions*:** The effectiveness of CCS-based PGS is comparable to that of traditional morphological methods, with better outcomes for women receiving IVF/ICSI technology. The transfer of both trophectoderm-biopsied and blastomere-biopsied CCS-euploid embryos can improve the implantation rate. | 2 |
| **INTRODUCTION** | | |  |
| Rationale | 3 | Aneupoidy is a very common abnormality in human embryos generated by IVF, especially for women with advanced maternal age (AMA). High percentage of aneuplodies has also been found in the embryos of women with repeated implantation failure (RIF), repeated pregnancy loss (RPL) and a partner with low sperm quality. The high frequency of aneuploidy and its likely deleterious effects on embryo viability, has led to the suggestion that embryos should be tested for chromosomal abnormalities before deciding on which ones to transfer to patients. For this purpose, preimplantation genetic screening (PGS) developed. FISH technology for selecting a panel of chromosomes used to be the most widely applied methodology for aneuploidy screening. But since previous data from random controlled trials with FISH-based PGS (PGS#1) showed no beneficial effect on live birth rates after IVF, and even lowered the live birth rate for women with AMA, the utilization of PGS#1 in attempts to improve IVF outcomes reduced worldwidely. However, the objective of comprehensive chromosome screening (CCS) is just assessing the whole chromosome complement (24 chromosomes). And several studies have been conducted to assess the impact of CCS-based PGS on IVF outcomes. | 4 |
| Objectives | 4 | To examine whether CCS-based PGS has an effect on improving IVF/ ICSI outcomes, we conducted a meta-analysis with eligible studies to assess the efficiency of CCS-based PGS for IVF/ ICSI outcomes compared to traditional morphological method in women with AMA, RIF, RPL or good prognosis. | 5 |
| **METHODS** | | |  |
| Protocol and registration | 5 | This meta-analysis has not been registered and there was no protocol existing. |  |
| Eligibility criteria | 6 | ***Type of studies***: Both RCTs and observational studies investigating the effects of CCS-based PGS were included without language and publication status limitations.  ***Type of participants***: Women more than 18 years old with AMA, RIF, RPL or good prognosis receiving IVF were included in this meta-analysis.  ***Types of intervention***: Trials comparing CCS-based PGS with traditional morphological method were included. We excluded trials associated with polar body biopsy, because polar body aneuploidy screening can only detect chromosomal abnormities of meitotic origin.  ***Types of outcome measures***: For all included studies, implantation rate per ET was defined as primary outcome, and secondary outcomes consisted of clinical pregnancy rate per cycle, ongoing pregnancy rate per cycle, live birh rate per cycle, miscarriage rate and multipregnancy rate. | 6 |
| Information sources | 7 | We conducted electronic searches in the database PubMed, Cochrane Library, CNKI (China National Knowledge Infrastructure) and *ClinicalTrials.gov* up to May 2015 with no study design limitations, and no language restriction. | 6 |
| Search | 8 | The following search terms were used: ‘preimplantation genetic diagnosis’ or ‘PGD’ or ‘preimplantation genetic screening’ or ‘preimplantation test’ or ‘screening for aneuploidies’ or ‘embryo selection’ or ‘embryo screening’ and ‘comprehensive chromosomal screening’ or ‘CCS’ or ‘array CGH’ or ‘array comparative genomic hybridization’ or ‘aCGH’ or ‘single nucleotide polymorphism’ or ‘SNP’ or ‘quantitative real-time PCR’ or ‘qPCR’ or ‘next-generation sequencing’ or ‘NGS’.  Search strategy: PubMed  01. preimplantation genetic diagnosis/  02. preimplantation genetic diagnosis. tw.  03. PGD/  04. PGD. tw.  05. preimplantation genetic screening/  06. preimplantation genetic screening. tw.  07. preimplantation test/  08. preimplantation test. tw.  09. screening for aneuploidies/  10. screening for aneuploidies. tw.  11. embryo selection/  12. embryo selection. tw.  13. embryo screening/  14. embryo screening. tw.  15. 1 or 2 or 3 or 4 or 5 or 6 or 7 or 8 or 9 or 10 or 11 or 12 or 13 or 14  16. comprehensive chromosomal screening/  17. comprehensive chromosomal screening. tw.  18. CCS/  19. CCS. tw.  20. array CGH/  21. array CGH. tw.  22. array comparative genomic hybridization/  23. array comparative genomic hybridization. tw.  24. aCGH/  25. aCGH. tw.  26. single nucleotide polymorphism/  27. single nucleotide polymorphism. tw.  28. SNP/  29. SNP. tw.  30. quantitative real-time PCR/  31. quantitative real-time PCR. tw.  32. qPCR/  33. qPCR. tw.  34. next-generation sequencing/  35. next-generation sequencing. tw.  36. NGS/  37. NGS. tw.  38. 16 or 17 or 18 or 19 or 20 or 21 or 22 or 23 or 24 or 25 or 26 or 27 or 28 or 29 or 30 or 31 or 32 or 33 or 34 or 35 or 36 or 37  39. 15 and 38  40. exp animals/ not humans | 6 |
| Study selection | 9 | After duplicate publications were removed, two authors (MH C and SY W) independently examined the possible relevant trials by checking the titles, abstracts and full-texts, and any problems of disagreement were resolved through group discussion. | 6 |
| Data collection process | 10 | We developed a data extraction form based on the Cochrane Concumers and Communication Review Group’s data extraction template and refined it accordingly. Two authors (MH C and SY W) independently extracted related information, and any problems of disagreement were resolved through group discussion. We didn’t contact authors for further information. | 6 |
| Data items | 11 | Information was extracted from included studies including: characteristics of included studies (including the first author’ name, study design, year of publication, study period, and trial’s inclusion and exclusion criteria), characteristics of included participants (including geographic region, sample sizes of groups, and indication for PGS), type of intervention (including type of CCS, day for biopsy, day for transfer, and fresh or frozen cycles), and type of outcome measurement (including implantation rate per ET, clinical pregnancy rate per cycle, ongoing pregnancy rate per cycle, live birh rate per cycle, miscarriage rate and multipregnancy rate). | 6 |
| Risk of bias in individual studies | 12 | Two reviewers (MH C and SY W) independently used the Newcatsle-Ottawa Scale (NOS) to assess the quality of included observational studies. And the Cochrane Collaboration’s Handbook was used to assess the quality of RCTs following the criterias: random sequence generation, allocation concealment, blinding, incomplete outcome data, selective outcome reporting and other potential sources of bias. | 7 |
| Summary measures | 13 | We calculated relative risk (RR) with corresponding 95% confidence intervals (CIs) for all outcomes reported in each study. | 8 |
| Synthesis of results | 14 | We identified the heterogeneity among studies bu conducting a standard Cochrane’s Q test with a significance level of α=0.10. And I^2^ statistic test was performed to examine the heterogeneity as well. I^2^≥50% was considered to indicate substantial heterogeneity. When heterogeneity existed, we attempted to figure out potential sources of heterogeneity by examining individual studies and subgroup analysis. Fixed-effect models were used to pool outcomes when heterogeneity among studies was considered to be statistically insignificant. Otherwise, random-effect model was used to combine the results. | 8 |
| Risk of bias across studies | 15 | Publication bias was estimated using Begg’s test. A value of “Pr > |z|” above 0.05 for Begg’s funnel plots was considered negative publication bias. | 8 |
| Additional analyses | 16 | Subgroup analysis was conducted according to study design, location, age of participants, indication for PGS, stage of biopsy, platform for CCS and number of embryos transferred. And one-way sensitivity analysis was performed to explore some factors that would influence the effects. | 8 |
| **RESULTS** | | |  |
| Study selection | 17 | A total of 1235 no duplicate titles and abstracts were identified in the initial search, and 23 articles were selected to undergo full-text assessment. Twelve studies did not fulfill the inclusion criteria. Finally, 4 RCTs and 7 cohort studies assessing the outcomes of CCS-based PGS versus traditional morphological-based selection in women undergoing IVF/ICSI met our inclusion criteria and were included in meta-analysis. The flow chart of the trials included in the meta-analysis is shown in Fig. 1. | 10 |
| Study characteristics | 18 | Main characteristics and quality features of the 4 RCTs and 7 cohort studies are shown in Table 1 and Table 2. | 10 |
| Risk of bias within studies | 19 | Study design and methodological quality varied among 4 RCTs. 1 study used a random number table to generate randomized sequence; 1 study used a computer-generated randomization; whereas, the randomization was stratified by age group in 1 study and the other one study did not explicitly describe sequence generation. Adequate measures of allocation concealment were used and explicitly described in only one study. Single blind was performed in one study; 1 study was not blinded, and the other 2 studies did not describe the method of blind. For the included 7 cohort studies, the NOS score ranges from 7 to 9, with a mean of 8 scores. All studies provided information on populations in the CCS group and the control group. But only 4 studies were well matched between CCS group and control group, and the other 3 studies were unmatched, so comparability bias might exist in the 3 studies for no control for important factors that could influence results. The follow up period for outcome was adequate for all studies, and outcome measurement was objective. | 21 |
| Results of individual studies | 20 | See Fig 2., Fig 3. and Table 3. | 21 |
| Synthesis of results | 21 | ***Implantation rate:*** Within 4 RCTs comparing CCS-based PGS and traditional morphological-based selection, the CCS group showed a higher implantation rate than the control group (RR 1.32, 95%CI 1.18-1.47). And the same effects were observed within 7 cohort studies (RR 1.74, 95%CI 1.35-2.24).  ***Clinical pregnancy*:** The outcome from pooled analysis for 2 RCTs showed a non-significant effect between the CCS group and the control group (RR 1.26, 95%CI 0.83-1.93). Whereas, a statistically significant effect of CCS-based PGS on increasing clinical pregnancy rate was observed in 6 cohort studies (RR 1.48, 95%CI 1.20-1.83).  ***Ongoing pregnancy*:** The pooled ongoing pregnancy rate in the CCS group seemed to be higher than the control group in 2 RCTs, but there was no significant difference between the two groups (RR 1.31, 95%CI 0.64-2.66). However, outcome from the pooled outcome of 5 cohort studies showed that CCS significantly improved the ongoing pregnancy rate (RR 1.61, 95%CI 1.30-2.00).  ***Live birth:*** In the RCT, there was a statistically significant increasing effect in live birth rate in the CCS group (61/72) compared with the control group (RR 1.26, 95%CI 1.05-1.50). But when the outcome was pooled for 3 cohort studies, no significant effect on live birth rate was observed between the CCS group and the control group (RR 1.35, 95%CI 0.85-2.13).  ***Miscarriage rate:*** The pooled outcome from 2 RCTs showed a decreased effect of miscarriage rate in the CCS group, but there was no significant difference between the two groups (RR 0.53, 95%CI 0.24-1.15). Nevertheless, the pooled analysis outcome including 5 cohort studies showed that the miscarriage rate was significantly lower in the CCS group (RR 0.31, 95%CI 0.21-0.46).  ***Multiple pregnancy:*** In the RCT, the multiple pregnancy rate was significantly lower in the CCS group (0/57) than control group (RR 0.02, 95%CI 0.00-0.26). Moreover, the same effect was observed in pooled outcome of 2 cohort studies (RR 0.19, 95%CI 0.07-0.51).  The heterogeneity in the pooled risk estimates of our outcomes ranged from an I^2^ test result of 0 to 90% for both RCTs and cohort studies. | 21 |
| Risk of bias across studies | 22 | The Begg’s test didn’t show significant small-study bias (p=0.062). | 25 |
| Additional analysis | 23 | Outcomes from subgroup analysis showed that implantation rate in the CCS group were higher than in the control group in any seperate subgroup. And the pooled effect results remained practically unchanged when we performed a one way sensitivity analysis. | 25 |
| **DISCUSSION** | | |  |
| Summary of evidence | 24 | CCS-based PGS was statistically significantly associated with imcreased implantation rate, which suggested that there was a great potential benefit of using CCS-based PGS over morphology method. Our results also fonud that CCS-based PGS was related to increased clinical pregnancy rate, ongoing pregnancy rate, live birth rate, decreased miscarriage rate and multipregnancy rate. | 28 |
| Limitations | 25 | Firstly, the quality of the studies varied among both RCTs and observational studies. Secondly, we also included a study presented just as an abstract. Thirdly, the indications for participants and the methods of CCS among studies were various. | 32 |
| Conclusions | 26 | The results indicate that selecting euploidy embryos by CCS technology may improve the IVF/ICSI outcomes for patients with good prognosis, as well as patients with AMA, RIF, and RPL. But our study provides no evidence on the benefit of CCS-based PGS for patients with poor ovarian reverse function or with a partner of low sperm quality, so further research is needed to confirm the effect of CCS for these patients. | 32 |
| **FUNDING** | | |  |
| Funding | 27 | No funding for this meta-analysis. |  |
